# Population Aging, Health Investment and Economic Growth: Based on a Cross-Country Panel Data Analysis

**DOI:** 10.3390/ijerph18041801

**Published:** 2021-02-12

**Authors:** Yingzhu Yang, Rong Zheng, Lexiang Zhao

**Affiliations:** 1 School of International Trade and Economics, University of International Business and Economics, Beijing 100029, China; 201700130004@uibe.edu.cn (Y.Y.); 201700160023@uibe.edu.cn (L.Z.); 2 World Health Organization Collaborating Center on Tobacco and Economics, Beijing 100029, China

**Keywords:** population aging, health investment, economic growth

## Abstract

With the economic development of various countries and the deepening of population aging, health plays an increasingly important role in the macro-economy. How to meet the growing health needs as well as promote the economy has captured the attention of the world. Therefore, whether health investment can promote economic growth is an important theoretical and practical issue. An extended Mankiw–Romer–Weil model (MRW) with human health capital and population aging is employed to examine the impact on economic growth from population aging and health investment. On the basis of the theoretical model, this paper uses the LSDV and TSLS methods to carry out an empirical study based on cross-country panel data during the period 2000–2016. The empirical results show that health investment plays a significant role in promoting economic growth, and there is an inverted U-shaped relationship between population aging and economic growth. The impacts on economic growth from health investment and population aging can weaken each other. In addition, this paper also finds that health investment structure and the proportion of government health investment to total government spending can affect economic growth.

## 1. Introduction

Population aging, caused by falling birth rate and increasing life expectancy, has become one of the most important challenges in the world. By world standard, a country or region enters the aging society when the number of people aged over 65 accounts for 7% of its total population [1]. In 2018, the number of people aged 65 and over unprecedentedly surpassed the number of people aged 0–4, at 705 million and 680 million, respectively, indicating a deepening trend of aging. Population aging is an inevitable result of social development. According to the UN’s projection, the proportion of the world’s population aged 65 and over will rise from 9% to 16% by 2050, which means one in six people will be over 65.

Population aging leads to significant changes in the age composition of the workforce and the overall population. With the deepening of aging population, the working-age population drops significantly, and human capital depreciation continues to accelerate. The demographic dividend gradually weakens or even disappears, which is undoubtedly an important reason for the slowdown of economic growth. Meanwhile, population aging also results in substantial changes in the total quality and age composition of human capital. Generally, as the workforce get older, their total working hours and labor productivity decrease. Additionally, the decrease of older and more experienced labor force far exceeds the increase of younger and well-educated labor force, which is not conducive to the accumulation of human capital. How to relieve the side effects caused by population aging has become a focus of governments and societies.

As an important force for sustainable economic growth, human capital has captured more and more attention. The endogenous growth theory emphasizes the important role of human capital accumulation on long-run economic growth. As two key components of human capital, education and health are vital for economic growth during the period of demographic transition. Most previous studies value education enough but ignore the role of health. However, human capital works only if the labor force is healthy. Thus, health expenditures affect economic growth by improving productivity and accumulation of human capital. Under the dual pressure of population aging and economic downturn, it is more important to improve health capital accumulation.

This paper integrates population aging, health investment, and economic growth into a unified model and uses international panel data to study the impact on economic growth from health investment and population aging. Research on these issues will provide a theoretical basis for the realization of sustainable and high-quality economic development with the background of population aging. It should be noted that there exists reverse causality between the core independent variables (health investment and population aging) and the dependent variable. To examine if the endogenous problem will affect the empirical results, this paper uses lag terms of health investment and population aging as instrumental variables and uses two-stage least square method (TSLS) to make empirical analysis. The rest of the paper is organized as follows. Section 2 presents the literature review and theoretical model. Section 3 introduces data and methodology. Section 4 presents empirical results and limitation of this paper. Section 5 gives the summary and conclusion of the study.

## 2. Materials and Methods

### 2.1. Literature Review

Many scholars pay attention to the impact of population aging on economic growth. Aging population has led to some huge economic consequences. The potential economic impacts of population aging are discussed in many papers. According to the existing research conclusions, the research about the economic impacts of population aging can be summarized into three categories: The first category of literature believes that population aging will have a negative impact on economic growth (Bloom et al., 2010 [2]; Daniele et al., 2019 [3]). This category of literature mainly demonstrates the negative effect of population aging on economic growth from the following three perspectives. First, the aging of the population will lead to a decrease in the proportion of the working-age population and labor force growth rate (Cutler et al., 1990 [4]). At the same time, the aging of the population will also change the overall productivity level of the society. Population aging can also indirectly hamper economic growth by raising labor costs (Cepar and Troha, 2015 [5]). Second, according to the life cycle theory, people have a high motivation to save when they are young. People have negative savings when they retire, so the deepening of population aging lowers the national savings rate (Heijdra and Ligthart, 2006 [6]; Auerbach and Kotlikoff, 1987 [7]; Auerbach et al., 1989 [8]; Miles, 1999 [9]; Hviding and Merette, 1998 [10]). The decrease in savings reduces the level of physical capital investment, which is decreases economic growth. The dual pressures of decreasing government revenue and increasing social security expenditures such as old-age pension and medical care will seriously restrict the government’s ability to guide investment. Third, population aging could lower interest rates. The capital will flow from countries with more aging to those with less aging (Liu and McKibbin, 2020 [11]).

The second category of literature argues that population aging has a beneficial impact on economic growth (Nagarajan et al., 2016 [12]; Maity and Sinha, 2020 [13]). First, population aging is caused by both the increase in life expectancy and the decrease in fertility rate. Although the decrease in fertility rate has a negative impact on economic growth, the increase in life expectancy has a positive impact on economic growth. The latter dominates the former (Prettner, 2013 [14]). Second, the aging of population improves the investment opportunities of human capital (Choi and Shin, 2015 [15]) and increases the years of schooling (Fougère and Mérette, 1999 [16]). As population aging extends life expectancy and reduces people’s preference for raising offspring (Ladd and Murray, 2001 [17]), people begin to pay more attention to the improvement of individual labor productivity instead of pursuing the increase of family labor force. To obtain higher labor remuneration in the future (Poterba, 1998 [18]), people increase their investment in education, technical training, and other fields to improve their and their family members’ skill level (Boucekkine et al., 2002 [19]; Gradstein and Kaganovich, 2004 [20]), which will promote technological progress. Then, population aging can also promote the upgrading of industrial structure (Boriss et al., 2011 [21]) and technological innovation through two other ways. The first is the learning by doing effect. With the increase of the average age of working population, experience and skill levels of work force increases, which improves the efficiency of innovation work. The second is that the labor shortage caused by population aging prompts the transformation of economic growth pattern and emphasis on technological progress, which in turn triggers the redistribution of social resources and promotes the technical progress and innovation in order to increase the economic merit (Lee and Mason, 2010 [22]).

The third category of literature considers that the relationship between population aging and economic growth is complex, uncertain, and nonlinear. In the study of the relationship between population age structure and economic growth in OECD countries, An and Jeon (2006) found that population aging and economic growth present an inverted U-shaped relationship [23]. Barro and Wolf (1989) pointed out that, when life expectancy increases from 60 to 69, the GDP per capita growth rises steadily. When the life expectancy exceeds 70, the GDP per capita growth rate declines but remains higher than in countries with low life expectancy [24]. Futagami and Nakajima (2001) explored how population aging affects economic growth by constructing an endogenous growth model with life-cycle savings. The results indicate that population aging is not necessarily bad for economic growth [25]. Liu Xiao-yong (2013) studied the influence of the population aging on the economic growth of provinces based on provincial panel data for 1989–2009 in China, and their research results suggest that there is an inverted U-shaped relationship between population aging and economic growth. The proportion of the elderly population has a decreasing positive effect on the inter-provincial economic growth rate, but, after crossing the inflection point, its effect changes from positive to negative [26].

The relationship between health investment and economic growth has been considered by many scholars for a long time, but no unified conclusion has been reached. In simple terms, the economic resources including labor and commodities used for healthcare comprise health investment. To some extent, health spending improves the labor productivity and continues to pay off for a long time (Mushkin, 1962 [27]). Government and residents are two main bodies of health investment. To meet people’s needs of high-quality medical and health services, the government increases investment in the medical establishment to improve the quality of medical service and increase medical resources (Castro, 2020 [28]). Residents invest in their health in the following ways: medical insurance, health checkup, fitness investment, health consultation, etc. Some scholars found that the economic growth rate is much higher than the increase rates of physical capital and labor force (Barro and Sala-i-Martin, 1995 [29]). Thus, they proposed the issue of “the impact on economic growth from population quality change”. Most papers discuss this issue from the viewpoint of human capital, and they argue that health investment promotes economic growth. As an investment, health determines the total amount of time that laborers can spend on economic and non-economic productive activities directly (Grossman, 1972 [30]). A healthy workforce would promote the human capital accumulation process. Human capital plays a much bigger role in economic growth than physical capital (Schultz, 1961 [31]), and its rate of return is also higher than physical capital. As an important part of capital accumulation, health investment can improve not only the productivity of individuals but also the productivity of the whole society (Lucas, 1989 [32]). Health investment could promote economic growth through the following two main ways: The first is that health investment could promote economic growth by improving labor force participation rate and production efficiency; health investment could improve individuals’ health status and individuals can work longer with better health and increased life expectancy (Bloom et al., 2009 [33]). The second is that health investment is conducive to human capital accumulation such as education and improves economic development efficiency by improving individual learning ability and returns to education (Fuchs, 1982 [34]). Others think that health investment blocks the economic growth because it will squeeze out physical investment, which is still the main contributing factor to economic growth. By analyzing the relationship between the composition of public expenditure and economic growth, Devarajan et al. (1996) found that health expenditure has a slightly negative impact on economic growth [35].

As discussed above, because of the decrease of working-age population caused by population aging, the first demographic dividend is wearing off. Given this, some thinks population aging will undermine economic development. On the contrary, other scholars believe that population aging can bring a second demographic dividend. Mason and Lee (2004) pointed out that low fertility and mortality will increase the capital–labor ratio (capital intensity) and output per capita [36]. The impact on economic growth from population aging is determined by the relative importance of capital over labor in production (Curtis and Lugauer, 2019 [37]). Lee and Mason (2006) believed that longer life expectancy will increase the consumption demand in old age, which will enhance people’s savings motive. In the perspective of the whole society, it will increase the accumulation of total social capital and promote economic growth [38]. Lee and Mason (2010) found out that population aging leads to a tremendous increase in education levels of the population. They thought human capital investment is a potentially important generation mechanism of a second demographic dividend [22]. Bairoliya et al. (2017) suggested that aging economies with high human capital may benefit from the increase of fertility [39].

Saving is an important mechanism by which population aging affects economic growth. It is necessary to discuss the impact on saving from changes in demographics. Some recent papers focus on the channels through which demographics affect saving. In general, there are four channels. First, as mentioned above, total savings increases with the proportion of working-age population. Second, as the life expectancy increases, people will save for more consumption after retiring (Bloom et al., 2007 [40]). Third, as the number of children declines, which leads to inadequate family insurance, people will increase precautionary savings. Fourth, as fertility decreases, some people who expect less retirement support from the next generation with smaller population will save more. Leff (1969) believed that there is a negative correlation between dependency ratio (including juvenile dependency ratio and old-age dependency ratio) and the household savings rate [41]. Curtis et al. (2017) thought that different demographic profiles could affect savings differently. In China and India, with relative younger population, the increase of household savings rate is mostly due to the rapid decline in the number of children. In Japan, the most elderly country in the word, the decrease of savings rate is partially caused by the growing number of retirees [42]. Imrohoroglu and Zhao (2018) suggested that the decline of family insurance caused by one-child policy in China increases savings rate [43].

### 2.2. The Model

We assume an agent’s utility depends on his consumption level and health level (Grossman, 1972 [30]). The level of health depends on the health investment in the adult life of representative individuals. The expected utility function of an agent is assumed as follows:

(1) $$\int_{0}^{+\infty }U\left(c_{t},h_{t}\right)e^{-(\rho -n)t}dt$$

with

(2) $$U\left(c_{t},h_{t}\right)=c_{t}^{\sigma }h_{t}^{1-\sigma }$$

where $c_{t}$ denotes consumption in period t, $h_{t}$ denotes health level in period t, and ρ≥0 is the rate of time preference, which measures the weight in utility attached to the health level.

The analysis is based on an extended Mankiw–Romer–Weil model with two kinds of human capital (Knowles and Owen, 1995 [44]). Output *Y* is produced as a function of total factor productivity *A*, physical capital *K*, labor input *L*, human education capital *E*, and human health capital *H*. At the same time, considering that population aging φ will affect labor supply, the production function is assumed to be:

(3) $$Y_{it}=K_{it}^{\alpha }E_{it}^{\beta }H_{it}^{\psi }\varphi_{it}{\left(A_{it}L_{it}\right)}^{1-\alpha -\beta -\psi },\;0\text{<}\alpha ,\beta ,\psi <1$$

Subscripts *i* and *t* denote countries and years, respectively. Dividing by the labor force *L_it_*, the per capita production function can be written as follows:

(4) $$y_{it}=\varphi_{it}k_{it}^{\alpha }e_{it}^{\beta }h_{it}^{\psi }$$

with $k_{it}=\frac{K_{it}}{A_{it}L_{it}}$ being capital per effective unit of labor, $e_{it}=\frac{E_{it}}{A_{it}L_{it}}$ human education capital per effective unit of labor, and $h_{it}=\frac{H_{it}}{A_{it}L_{it}}$ human health capital per effective unit of labor. We assume population and technology grow with fixed rates n and g, respectively. Then, the accumulation equations of the three kinds of capital are as follows:

(5) $$\;\;\;\;\;\;{\dot{k}}_{it}=\varphi_{it}s_{ki}y_{it}-\left(n_{it}+g+\delta \right)k_{it}$$

(6) $${\dot{e}}_{it}=s_{ei}y_{it}-\left(n_{it}+g+\delta \right)e_{it}$$

(7) $$\;{\dot{h}}_{it}=s_{hi}y_{it}-\left(n_{it}+g+\delta \right)h_{it}$$

where $s_{ki}$,$\;s_{ei}$, and $s_{hi}$ denote the accumulation rate of physical capital, human education capital, and human health capital respectively, and $\delta_{ki}$,$\;\delta_{ei}$, and $\delta_{hi}$ denote their respective depreciation rates.

Each agent maximizes his own utility by selecting consumption and the levels of the accumulation of physical capital, human education capital, and human health capital. Therefore, the optimization problem can be written as follows:

$$max\int_{0}^{+\infty }U\left(c_{t},h_{t}\right)e^{-(\rho -n)t}dt$$

$$s.t.\;\dot{k}=\varphi s_{k}y-\left(\delta_{k}+n+g\right)k$$

$$\dot{e}=s_{e}y-\left(\delta_{e}+n+g\right)e$$

$$\dot{h}=s_{h}y-\left(\delta_{h}+n+g\right)h$$

$$\;\;\;\;c=\left(1-s_{k}-s_{e}-s_{h}\right)y$$

Then, we assume the existence of a steady state (with α+β+ψ<1). To simplify the analysis, we assume δ is the common depreciation rate. The Hamiltonian function is as follows:

$$H=c_{t}^{\sigma }h_{t}^{1-\sigma }+\lambda_{k}\left[\varphi s_{k}y-\left(\delta +n+g\right)k\right]+\lambda_{e}\left[s_{e}y-\left(\delta +n+g\right)e\right]+\lambda_{h}\left[s_{h}y-\left(\delta +n+g\right)h\right]$$

According to the dynamic equilibrium condition ($\dot{k}=\dot{e}=\dot{h}=\dot{c}={\dot{\lambda }}_{k}={\dot{\lambda }}_{e}={\dot{\lambda }}_{h}=\dot{\mu }=0$), we can get steady-state values, which are denoted by asterisks:

(8) $$k_{i}^{*}={\left(\frac{\varphi_{i}^{2-\beta -\psi }s_{ki}^{1-\beta -\psi }s_{ei}^{\beta }s_{hi}^{\psi }}{n_{i}+g+\delta }\right)}^{\frac{1}{\eta }}$$

(9) $$\;e_{i}^{*}={\left(\frac{\varphi_{i}^{1+\alpha }s_{ki}^{\alpha }s_{ei}^{1-\alpha -\psi }s_{hi}^{\psi }}{n_{i}+g+\delta }\right)}^{\frac{1}{\eta }}$$

(10) $$\;h_{i}^{*}={\left(\frac{\varphi_{i}^{1+\alpha }s_{ki}^{\alpha }s_{ei}^{\beta }s_{hi}^{1-\alpha -\beta }}{n_{i}+g+\delta }\right)}^{\frac{1}{\eta }}$$

and η=1−α−β−ψ. According to Equations (4)–(7), and taking the natural logs. Then, we can get following equation:

(11) $$ln\left(\frac{Y_{it}}{L_{it}}\right)=A_{0}+\frac{1+\alpha }{1-\eta }ln\left(\varphi_{it}\right)+gt-\frac{1-\eta }{\eta }ln{\left(n_{i}+g+\delta \right)}_{t}+\frac{\alpha }{\eta }ln\left(s_{ki}\right)+\frac{\beta }{\eta }ln\left(s_{ei}\right)+\frac{\psi }{\eta }ln\left(s_{hi}\right)\;$$

### 2.3. Data and Methodology

Based on the theoretical model above, we set the basic econometric model as follows:

(12) $$g_{it}=\alpha_{0}+\alpha_{1}H_{it}+\alpha_{2}A_{it}+\alpha_{3}E_{it}+\alpha_{4}K_{it}+\alpha_{5}n_{it}+\varepsilon_{it}\;$$

where subscripts *i* and *t* denote countries and years, respectively. g refers to economic growth measured by logarithmic form of GDP per capita, health capital H is measured by the ratio of total health expenditure to GDP, A refers to population aging measured by proportion of population aged 65 and over, educational capital E is measured by primary student-to-teacher ratio, K refers to accumulation rate of physical capital measured by ratio of gross domestic fixed capital formation to GDP, n refers to population growth rate, and ε is the stochastic disturbance term.

To further study the interaction between population aging and health investment on economic growth, this paper introduces the interaction term between population aging and health investment (HEAA) into the basic regression model, as shown in Equation (13):

(13) $$g_{it}=\alpha_{0}+\alpha_{1}H_{it}+\alpha_{2}A_{it}+\alpha_{3}E_{it}+\alpha_{4}K_{it}+\alpha_{5}n_{it}+\alpha_{6}HEAA_{it}+\varepsilon_{it}\;$$

To examine the impact on economic growth from health investment structure and financial expenditure structure, the two variables are added in the model (Equation (14)):

(14) $$g_{it}=\alpha_{0}+\alpha_{1}H_{it}+\alpha_{2}A_{it}+\alpha_{3}E_{it}+\alpha_{4}K_{it}+\alpha_{5}n_{it}+\alpha_{6}HS_{it}+\alpha_{7}FS_{it}+\varepsilon_{it}\;$$

*HS* refers to health investment structure measured by the proportion of government health investment to total health investment and *FS* refers to financial expenditure structure measured by the proportion of government health investment to total government spending.

Using panel data from 186 countries and regions from 2000 to 2016, this paper empirically investigates the impact on economic growth of health investment and population aging.

The data for empirical analysis were obtained from a variety of sources. The per capita GDP and ratio of gross domestic fixed capital formation to GDP were obtained from the World Bank’s national accounts database, the OECD national accounts database, and WDI world bank development indicators database. World Health Organization (WHO) (World Health Organization 2005 [1]) statistics were the source for population growth, the total population, the number people over 65 years old, and old-age dependency ratio. Primary student-to-teacher ratio was obtained from UNESCO Institute of Statistics. The ratio of health expenditure to GDP was derived from WHO Global Health Expenditure database. The ratio of people over 65 years old was calculated by dividing the number of people over 65 years old by the total population.

**Figure 1:**
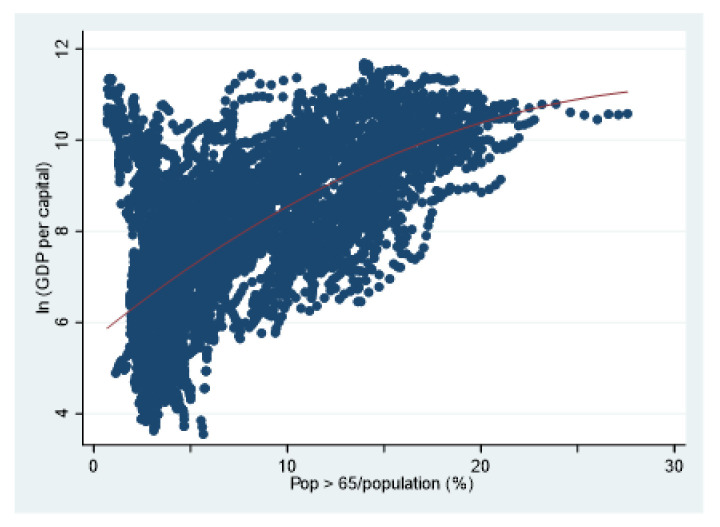
Population aging and economic growth.

Summary statistics of the sample data are presented in Table 1. Table 2 presents pairwise correlation. Health investment and economic growth are positively correlated, which is consistent with the traditional human capital theory. Population aging and economic growth also present a positive relationship. Following Liu (2013) [26], this paper uses a scatter plot to observe the relationship between population aging and economic growth. Through the scatter plot, the relationship between population aging and economic growth can be intuitively seen. Figure 1 shows that aging population and economic growth present a tendency of positive correlation, but the positive relationship is gradually weakened. This is probably because, in the initial stage, the acceleration of physical capital accumulation and the extension of demographic dividend brought by population aging are conducive to economic growth, but, with the deepening of population aging, the positive relationship between population aging and economic growth gradually weakens and may even change.

## 3. Results

### 3.1. Estimation Results of Basic Model

The Hausman test proves the fixed effect model is superior to the random effect model (see Table 3), so the paper uses fixed effect mode for data analysis. Meanwhile, in view of the characteristics of transnational panel data, this paper uses LSDV method to estimate the fixed effect model to eliminate the bias of estimation results caused by individual differences. The regression results are shown in Table 3. Column (1) shows that health expenditure plays a promoting role in economic growth. A 1% increase in the ratio of health expenditure to GDP is accompanied by an increase in per capita GDP of 0.067%.

As shown in Figure 1, there may be a nonlinear relationship between population aging and economic growth. Thus, Column (2) adds the quadratic term of population aging to examine this. The result shows that the coefficient of the primary term of population aging is significantly positive and the coefficient of the quadratic term is significantly negative, which indicates that the relationship between population aging and economic growth presents an inverted U-shaped pattern. At first, the increase of the ratio of the elderly population can promote economic growth. However, with the deepening of population aging, its impact on economic growth gradually becomes negative. This is consistent with previous research results [23,26].

Column (3) puts health investment and population aging into the regression at the same time. The regression results are basically consistent with Columns (1) and (2). The coefficient of health investment and the primary term of population aging are significantly positive. The coefficient of the quadratic term of population aging is significantly negative. However, the absolute value of the coefficient is slightly smaller.

Column (4) controls variables affecting economic growth such as the ratio of gross domestic fixed capital formation to GDP, primary student-to-teacher ratio, and population growth, rate and the regression results are still robust. The accumulation of education capital and physical capital and population growth could contribute to economic growth.

In view of endogenous problem, this paper uses the lag terms of health investment and population aging as the instrumental variables (Wang et al., 2017 [45]) and uses two-stage least square method (TSLS) to estimate the above basic model. The estimation results are shown in Table 3.

The significance and signs of the regression coefficients of the core variables health (investment, population aging, and the square term) are all consistent with the results estimated by the LSDV method. The absolute value of coefficients is a little bigger than the LSDV estimation results. The above conclusions still exist after considering the endogenous problem by instrumental variable method.

### 3.2. Heterogeneity Analysis

Then, a further analysis is made using data from 40 European countries, and the results are shown in Table 4. Both the LSDV method and the TSLS method are used, and the regression results are robust. The significance and sign of core explanatory variables are basically consistent with the full sample regression. However, the absolute value of coefficients is larger, which means the effect of both health investment and population aging on economic growth are stronger in European countries.

### 3.3. Robustness Check

Then, this paper uses the old-age dependency ratio as an alternative index of the population aging to test the robustness of the inverted U-shaped relationship between population aging and economic growth. The old-age dependency ratio is equal to the number of people aged 65 and over compared to the number of people of working age aged 15–64. In Table 5, the regression results shown in Columns (1) and (2) were obtained by LSDV and TSLS, respectively. Compared with the basic regression results, the regression results are very robust.

### 3.4. Interaction between Population Aging and Health Investment on Economic Growth

According to the above research results, health investment contributes to economic growth, and the relationship between population aging and economic growth is inverted U-shaped. Therefore, it is one-sided to consider the impact of the two on economic growth in isolation. It is of great theoretical and practical significance to consider the interaction of the two on economic growth. Thus, to further study the interaction between population aging and health investment on economic growth, this paper performs a regression analysis based on Equation (13).

LSDV and TSLS were used for regression, respectively. The estimation results are shown in Table 6. As shown in Table 6, the coefficients of health investment and population aging are significantly positive, and the coefficient of the quadratic term of population aging is significantly negative, which are consistent with the basic regression results above. The coefficient of interaction is significantly negative, indicating the effects of health investment and population aging on economic growth could weaken each other. As shown in Column (2), the TSLS method was used to estimate endogeneity, and the estimated results are consistent with those of the LSDV method. That means that, when population aging hinders economic growth, health investment can compensate the negative effect, while, when population aging promotes economic growth, the health investment would weaken the positive effect.

In the initial stage of population aging, the increase of elderly population proportion has a promoting effect on economic growth, while the increase of health investment will squeeze out physical capital investment, consumption, and other factors conductive to economic growth. At this moment, because the aging population proportion is still relatively small and the payout of health investment income is not obvious, the crowding-out effect of health investment is greater than the accumulation effect of health capital. Thus, health investment will weaken population aging’s promoting effect on economic growth. However, with the deepening of population aging, problems such as lack of labor force and increase of dependency burden begin to hinder the economic growth. At this point, the capital accumulation effect of health investment gradually outweighs its crowding-out effect, which is conducive to the accumulation of human capital in the whole society and the transformation from demographic dividend to talent dividend. Thus, the increase of health investment helps to weaken the negative effect of population aging on economic growth.

### 3.5. Further Study

We examine the effect of absolute level of health investment on economic growth. Generally, the total health investment composes of government health investment and private health investment. There is a proportion between them; according to the principle of diminishing marginal returns, this proportion may affect economic growth as well. For the government, health investment may crowd out physical capital investment. As the government is the main body of health investment, the financial expenditure structure may affect economic growth too. In addition to the total amount of health investment, does the structure of health investment and fiscal expenditure affect the economic growth effect of health investment? If the answer is yes, how do they affect economic growth? To study the above problems, this paper makes empirical analysis on basis of Equation (14).

On the basis of Equation (14), this paper adds HS’s interaction term with government health investment (HS#GOVHEA) into the model to study if and how the health investment structure influences the effect of government health investment on economic growth. This paper also adds the quadratic terms of *HS* (HS2) and *FS* (FS2) into the model, respectively, to examine if there is a nonlinear relationship between them and economic growth. Then, this paper uses the LSDV method to estimate the results.

As shown in Table 7, both the signs and significance of the core explanatory variables’ coefficients are relatively robust and almost unchanged compared with the results of the basic regression. In Column (1), two variables, namely health investment structure and financial expenditure structure, are added. The coefficient of the former variable is significantly positive, while the coefficient of the latter is significantly negative. In Column (2), the quadratic term of health investment structure is added. The result shows that the coefficient of health investment structure is significantly positive and the coefficient of quadratic term of significantly negative. Thus, the relationship between health investment structure and economic growth is inverted U-shaped. This shows that there is an optimal proportion between government health investment and private health investment. When the proportion is relatively small, the faster growth of government health investment compared with private health investment will lead to an increase in per capita GDP. When the proportion is higher than the optimal proportion, the faster growth of government health investment compared with private health investment will hinder the growth of per capita GDP. In Column (3), the financial expenditure structure and its quadratic term are added on the basis of Column (2). The regression coefficient of financial expenditure structure is significantly negative, while the coefficient of the quadratic term is significantly positive. This means that there is a U-shaped relationship between financial expenditure structure and economic growth. This indicates that, when the accumulation rate of healthy human capital is low, the crowding out effect on physical capital is larger than the effects of investment and accumulation of human capital, so the increase of the proportion of government health investment to total government spending may have a negative impact on economic growth. When the accumulation rate of healthy human capital exceeds a certain proportion, the effects of investment and accumulation of human capital are bigger. At this moment, the increment of this proportion plays a promoting role in economic growth. In Column (4), health investment structure’s interaction term with government health investment is added into the regression. The regression coefficient of the interaction term is significantly negative, which indicates the proportion of government health investment to total health investment will weaken government health investment ‘s positive effect on economic growth. In other words, the marginal effect of government health investment on economic growth diminishes as the proportion of government health investment to total health investment increases.

## 4. Discussion

With the development of economy and society, people’s fertility desire declines and life expectancy improves, which means that population aging has gradually become a characteristic of global demographic change (Judith et al., 2013 [46]). How to deal with the aging population is a matter of cardinal significance faced by all countries. The changes of aging groups have a wide and profound influence on various aspects of social life in the new century. Presently, all developed countries and some developing countries have entered the aging society, and more developing countries will enter the aging society soon. Many countries such as Japan and Italy have experienced the deepening of aging population degree and slowdown of economic growth (Luděk et al., 2020 [47]; Mitra and Abedin, 2020 [48]). Population aging is likely to be one of the important factors leading to the decline of economic growth in these countries. To cope with the problem of population aging, many countries have begun to increase the health investment. To examine the impact of population aging on economic growth and whether health investment can influence their relationship, the panel data of 186 countries and regions are analyzed using the LSDV and TSLS methods. Most of the existing literature tests the impact of population aging and health investment on economic growth, respectively. Few papers study the impact of population aging on economic growth from the perspective of health investment. Few scholars study population aging, health investment, and economic growth within the same framework. This paper not only puts population aging, health investment, and economic growth under the same analytical framework but also discusses how health investment structure and the proportion of government health investment to total government spending affect economic growth. All of these make up for the lack of mainstream literature in this respect at present as well as provide a new perspective for the future study.

The regression results show that health investment has a significant positive impact on economic growth, which is consistent with the health-led growth hypothesis (HLGH). Many studies support this conclusion, including those by Lewis and Jack (2009), Mehrara and Musai (2011), Sülkü and Caner (2011), Wang (2011), Atilgan et al. (2017) and Adel and Imène (2019) [49,50,51,52,53,54]. The second conclusion is that there is a significant inverted U-shaped relationship between population aging and economic growth. This conclusion means that the economic growth rate increases during the early stage of population aging and then decreases with the deepening of population aging. This result is supported by the research of An and Jeon (2006) [23] and Liu (2013) [26]. Another important finding from the study is that health investment would weaken the effect of population aging on economic growth, which is consistent with the study by He et al. (2015) [55]. Our findings also show that the increase of health investment promotes economic growth, but the positive effect of health investment is influenced by financial expenditure structure. To some extent, this result accords with those of Pecchenino and Pollard (2002) [56], who argued excessive health expenditure will squeeze out education expenditure. Meanwhile, there exists an inverted U-shaped relationship between health investment structure and economic growth. It means that it is worth increasing health investment for government until the proportion of government health investment to private investment reaches an optimal value. These points are the innovations of this paper.

The paper is not without limitations. Limited by the data available, the empirical analysis uses health expenditure/GDP to measure health investment without distinguishing different types of health investment. The economic impact from different types of health investment may be different. Different types of health investment would have different effects on impact on economic growth from population aging. Future research could discuss the impact on economic growth from different types of health investment and their influences on impact on economic growth from population aging.

## 5. Conclusions

This paper uses cross-country panel data to examine the effects of population aging and health investment on economic growth. The results of the basic model suggest that health investment can promote economic growth and that there is an inverted U-shaped relationship between population aging and economic growth. Another major conclusion of this paper is that the effects of health investment and population aging on economic growth can weaken each other. The results of further study show two other conclusions. The first is that there is an optimal value of health investment structure, which is measured by the proportion between government health investment and private health investment. The second is that the proportion of government health investment to total health investment will weaken the marginal effect of government health investment on economic growth.

## Figures and Tables

**Table 1:** Summary statistics of sample data.

Variable Name	Variable Definition	Label	N	Mean	S.D.	Min	Max
Economic growth	ln(GDP per capita)	g	9091	7.61	1.73	3.549	11.685
Health investment	Health expenditure/GDP (%)	HEA	3132	6.06	2.28	1.025	25.475
Population aging	Pop >65/population (%)	AGE	9091	8.02	5.56	0.686	27.576
	old-age dependency ratio	age	9091	10.42	6.53	0.796	46.171
Physical capital investment	domestic fixed capital formation/GDP (%)	INV	1959	23.01	7.26	2.781	68.023
Education capital stock	primary student-to-teacher ratio	PTRATIOP	1959	26.07	14.23	8.141	100.237
Population growth	Population growth	POP	1959	1.39	1.40	−3.848	15.177
Government health investment	Government health expenditure/GDP (%)	GOVHEA	1959	3.26	2.30	0.105	13.780
Health investment structure	government health investment/total health investment (%)	HS	1959	51.04	22.22	3.916	95.026
Financial expenditure structure	government health investment/total government spending (%)	FS	1959	9.73	4.79	0.611	36.643

Note: The real GDP per capita is in 2000 constant prices.

**Table 2:** Pairwise correlation.

Variables	g	HEA	AGE	INV	PTRATIOP	POP
g	1.000					
HEA	0.230 *	1.000				
AGE	0.661 *	0.494 *	1.000			
INV	0.209 *	−0.171 *	0.074 *	1.000		
PTRATIOP	−0.726 *	−0.213 *	−0.588 *	−0.115 *	1.000	
POP	−0.295 *	−0.276 *	−0.593 *	−0.065 *	0.347 *	1.000

Note: Asterisks indicate significance levels: * *p* < 0.01.

**Table 3:** Basic model estimation results.

	(1)	(2)	(3)	(4)	(6)	(7)
	LSDV	LSDV	LSDV	LSDV	TSLS	TSLS
HEA	0.066 ***	-	0.036 ***	0.057 ***	0.042 ***	0.084 ***
	(0.013)	-	(0.010)	(0.012)	(0.011)	(0.013)
AGE	-	0.719 ***	0.490 ***	0.332 ***	0.483 ***	0.320 ***
	-	(0.015)	(0.027)	(0.033)	(0.030)	(0.033)
AGE2	-	−0.014 ***	−0.010 ***	−0.006 ***	−0.010 ***	−0.006 ***
	-	(0.001)	(0.001)	(0.001)	(0.001)	(0.001)
INV	-	-	-	0.017 ***	-	0.015 ***
	-	-	-	(0.003)	-	(0.002)
PTRATIOP	-	-	-	−0.044 ***	-	−0.043 ***
	-	-	-	(0.004)	-	(0.003)
POP	-	-	-	0.035 **	-	0.040 ***
	-	-	-	(0.015)	-	(0.013)
Constant	5.330 ***	4.677 ***	4.528 ***	6.380 ***	5.223 ***	6.454 ***
	(0.176)	(0.141)	(0.155)	(0.274)	(0.150)	(0.210)
Observation	3132	9091	3003	1959	2825	1848
Hausman	3.29 *	245.76 ***	39.82 ***	14.37 **		
F statistics	192.110 ***	101.790 ***	126.30 ***	201.940 ***	129.450 ***	69.400 ***
Kleibergen–Paap rk LM statistic					205.137 ***	164.112 ***
Kleibergen–Paap rk Wald F statistic					324.756	209.146
R^2^	0.928	0.825	0.938	0.955	0.433	0.603

Note: (1) Standard errors in parentheses. (2) Asterisks indicate significance levels: * *p* < 0.1, ** *p* < 0.05, *** *p* < 0.01.

**Table 4:** Heterogeneity analysis: Samples from 40 European countries.

	(1)	(2)
	LSDV	TSLS
HEA	0.087 ***	0.110 ***
	(0.018)	(0.023)
AGE	0.555 ***	0.563 ***
	(0.077)	(0.085)
AGE2	−0.011 ***	−0.011 ***
	(0.002)	(0.002)
INV	0.041 ***	0.037 ***
	(0.004)	(0.005)
PTRATIOP	−0.041 ***	−0.031 ***
	(0.011)	(0.011)
POP	0.097 ***	0.115 ***
	(0.033)	(0.034)
Constant	2.645 ***	2.380 ***
	(0.727)	(0.798)
Observation	520	493
Hausman	287.550 ***	
F statistics	166.740 ***	50.220 ***
Kleibergen–Paap rk LM statistic		52.747 ***
Kleibergen–Paap rk Wald F statistic		151.079
R^2^	0.941	0.466

Note: (1) Standard errors in parentheses. (2) Asterisks indicate significance levels: *** *p* < 0.01.

**Table 5:** Robustness test: Old-age dependency ratio.

	(1)	(2)
VARIABLES	LSDV	TSLS
HEA	0.078 ***	0.112 ***
	(0.010)	(0.014)
age	0.110 ***	0.081 ***
	(0.020)	(0.021)
age2	−0.001 ***	−0.001 *
	(0.000)	(0.000)
INV	0.018 ***	0.015 ***
	(0.002)	(0.002)
PTRATIOP	−0.052 ***	−0.051 ***
	(0.003)	(0.003)
POP	0.029 **	0.034 ***
	(0.013)	(0.013)
Constant	6.793 ***	7.653 ***
	(0.225)	(0.217)
Observation	1959	1848
Hausman	373.980 ***	
F statistics	66.020 ***	81.460 ***
Kleibergen–Paap rk LM statistic		163.207 ***
Kleibergen–Paap rk Wald F statistic		215.240
R^2^	0.951	0.614

Note: (1) Standard errors in parentheses. (2) Asterisks indicate significance levels: * *p* < 0.1, ** *p* < 0.05, *** *p* < 0.01.

**Table 6:** Interaction between population aging and health investment on economic growth.

	Full Sample		Samples from 40 European Countries	
	(1)	(2)	(3)	(4)
	LSDV	TSLS	LSDV	TSLS
HEA	0.085 ***	0.144 ***	0.410 ***	0.531 ***
	(0.019)	(0.022)	(0.061)	(0.073)
AGE	0.339 ***	0.331 ***	0.585 ***	0.585 ***
	(0.034)	(0.033)	(0.075)	(0.083)
AGE2	−0.005 ***	−0.003 ***	−0.005 **	−0.004
	(0.001)	(0.001)	(0.002)	(0.003)
INV	0.017 ***	0.014 ***	0.040 ***	0.038 ***
	(0.003)	(0.002)	(0.004)	(0.004)
PTRATIOP	−0.045 ***	−0.044 ***	−0.035 ***	−0.024 **
	(0.004)	(0.003)	(0.011)	(0.011)
POP	0.036 **	0.042 ***	0.116 ***	0.133 ***
	(0.015)	(0.013)	(0.033)	(0.033)
HEAA	−0.004 **	−0.009 ***	−0.024 ***	−0.031 ***
	(0.002)	(0.003)	(0.004)	(0.005)
Constant	6.212 ***	6.283 ***	−0.024 ***	−0.031 ***
	(0.294)	(0.216)	(0.004)	(0.005)
Observation	1959	1848	520	493
Hausman	116.910 ***		1538.400 ***	
F statistics	226.960 ***	99.330 ***	88.480 ***	40.380 ***
Kleibergen–Paap rk LM statistic		94.180 ***		84.257 ***
Kleibergen–Paap rk Wald F statistic		83.400		198.601
R^2^	0.955	0.589	0.944	0.494

Note: (1) Standard errors in parentheses. (2) Asterisks indicate significance levels: ** *p* < 0.05, *** *p* < 0.01.

**Table 7:** Health investment structure, financial expenditure structure, and economic growth.

	(1)	(2)	(3)	(4)
	LSDV	LSDV	LSDV	LSDV
HEA	0.081 ***	0.055 ***	0.092 ***	-
	(0.012)	(0.010)	(0.011)	-
GOVHEA	-	-	-	0.437 ***
	-	-	-	(0.049)
AGE	0.340 ***	0.329 ***	0.352 ***	0.326 ***
	(0.031)	(0.031)	(0.031)	(0.031)
AGE^2^	−0.006 ***	−0.006 ***	−0.007 ***	−0.006 ***
	(0.001)	(0.001)	(0.001)	(0.001)
INV	0.016 ***	0.017 ***	0.015 ***	0.016 ***
	(0.002)	(0.002)	(0.002)	(0.002)
PTRATIOP	−0.043 ***	−0.044 ***	−0.041 ***	−0.043 ***
	(0.003)	(0.003)	(0.003)	(0.003)
POP	0.042 ***	0.031 **	0.038 ***	0.040 ***
	(0.013)	(0.013)	(0.013)	(0.012)
HS	0.007 ***	0.018 ***	0.036 ***	0.004 **
	(0.002)	(0.004)	(0.005)	(0.002)
HS^2^	-	−0.000 ***	−0.000 ***	-
	-	(0.000)	(0.000)	-
FS	−0.028 ***	-	−0.092 ***	−0.040 ***
	(0.007)	-	(0.017)	(0.008)
FS^2^	-	-	0.002 ***	-
	-	-	(0.001)	-
HS#GOVHEA	-	-	-	−0.004 ***
	-	-	-	(0.001)
Constant	-	6.337 ***	5.917 ***	6.730 ***
	-	(0.212)	(0.223)	(0.198)
Observation	1959	1959	1959	1959
Hausman	175.240 ***	149.480 ***	155.400 ***	225.780 ***
F statistics	128.510 ***	129.370 ***	108.550 ***	120.670 ***
R^2^	0.951	0.955	0.956	0.956

Note: (1) Standard errors in parentheses. (2) Asterisks indicate significance levels: ** *p* < 0.05, *** *p* < 0.01.

## Data Availability

Not applicable.
